# Nutrients, Microglia Aging, and Brain Aging

**DOI:** 10.1155/2016/7498528

**Published:** 2016-01-31

**Authors:** Zhou Wu, Janchun Yu, Aiqin Zhu, Hiroshi Nakanishi

**Affiliations:** ^1^Department of Aging Science and Pharmacology, Faculty of Dental Science, Kyushu University, Fukuoka 812-8582, Japan; ^2^Department of General Surgery, Peking Union Medical College Hospital, Chinese Academy of Medical Sciences, Beijing 100730, China; ^3^Institution of Geriatric Qinghai Provincial Hospital, Xining 810007, China

## Abstract

As the life expectancy continues to increase, the cognitive decline associated with Alzheimer's disease (AD) becomes a big major issue in the world. After cellular activation upon systemic inflammation, microglia, the resident immune cells in the brain, start to release proinflammatory mediators to trigger neuroinflammation. We have found that chronic systemic inflammatory challenges induce differential age-dependent microglial responses, which are in line with the impairment of learning and memory, even in middle-aged animals. We thus raise the concept of “microglia aging.” This concept is based on the fact that microglia are the key contributor to the acceleration of cognitive decline, which is the major sign of brain aging. On the other hand, inflammation induces oxidative stress and DNA damage, which leads to the overproduction of reactive oxygen species by the numerous types of cells, including macrophages and microglia. Oxidative stress-damaged cells successively produce larger amounts of inflammatory mediators to promote microglia aging. Nutrients are necessary for maintaining general health, including the health of brain. The intake of antioxidant nutrients reduces both systemic inflammation and neuroinflammation and thus reduces cognitive decline during aging. We herein review our microglia aging concept and discuss systemic inflammation and microglia aging. We propose that a nutritional approach to controlling microglia aging will open a new window for healthy brain aging.

## 1. Introduction

The cognitive decline associated with aging and Alzheimer's disease (AD) will be a major issue in aging societies around the world as the life expectancy continues to increase. A better understanding of the factors that accelerate this cognitive decline will help in the development of strategies for preventing or delaying this cognitive decline. Microglia, the resident mononuclear phagocytes in the brain, are chronically or pathologically activated to influence the neuronal environment. There is increasing evidence that activated microglia produce excessive reactive oxygen species (ROS) during aging [1] and hypoxia [2–6], resulting in the nuclear factor-*κ*B- (NF-*κ*B-) dependent excessive production of proinflammatory mediators, including interleukin-1*β* (IL-1*β*), tumor necrosis factor-*α* (TNF-*α*), and interleukin-6 (IL-6) [7–11]. Furthermore, activated microglia-mediated neuroinflammation is closely associated with the pathogenesis of AD pathogenesis [12], because activated microglia trigger neuroinflammation to promote neuronal damage and the deposition of amyloid *β* (A*β*) [13, 14]. Moreover, anti-inflammatory agents improve the cognitive functions of AD patients [15, 16].

It is well accepted that chronic systemic inflammation can alter the neuroinflammation in the brain [17, 18]. In addition to being associated with systemic diseases such as atherosclerosis and diabetes, rheumatoid arthritis (RA), periodontitis, and inflammatory bowel disease (IBD) also directly initiate or hasten the progression of AD [19]. A clinical study has demonstrated the impact of RA and periodontitis on AD [20], and recent experimental studies have clarified the routes of inflammatory signal transduction from chronic systemic inflammation to the brain [17, 18, 20].

We have recently found that natural products, such as propolis, inhibit the hypoxia-induced production of proinflammatory mediators by microglia through the inhibition of mitochondria-derived ROS generation and the subsequent activation of the NF-*κ*B signaling pathway. Furthermore, we have found that RNSP, a traditional Tibetan medicine which is composed of 70 herbal components, improves the cognitive function in middle-to-moderate AD patients living at high altitude by reducing the levels of proinflammatory mediators and the deposition of A*β* [21]. In the present review, we will highlight our proposed concept of microglia aging, which refers to the fact that microglia are the potent accelerators of brain aging due to their induction of cognitive decline. We will also discuss the benefits of nutrients in preventing microglia aging and cognitive decline.

## 2. The Risk of Systemic Inflammatory Diseases for AD

RA is a chronic inflammatory bone disorder, which causes joint damage. A postmortem survey found that the prevalence of AD was reduced in RA patients who were long-term users of nonsteroidal anti-inflammatory agents [22–25]. More recently, patients with midlife RA were confirmed to have an increased risk of cognitive impairment, over a 21-year follow-up study, in several case-control and hospital- and register-based studies that were performed to examine the association between RA/arthritis and dementia/AD [26].

Periodontitis is a chronic inflammatory disorder in the periodontal tissues. There is growing clinical evidence to support a close link between periodontitis and the development and progression of AD [27, 28]. More recently, the three major periodontal bacteria, “Red complex,” including* Treponema denticola*,* Tannerella forsythia*, and* Porphyromonas gingivalis*, and their components have been detected in the brain of AD patients [29, 30]. More details have been reviewed by us recently [20].

IBD is a chronic inflammatory disorder in the gut. The gut bacteria are important for inducing systemic inflammation, and LPS is a potentially associated mediator which migrates into the intestinal capillaries [31]. Indeed, elevated LPS concentrations can be found in the plasma of AD patients [32–34], which supports a possible role of LPS in the promotion of neuroinflammation, and the triggering cognitive decline [35–37]. Furthermore, the chronically inflamed gut generates systemic proinflammatory cytokines to promote neuroinflammation, which causes cognitive decline [38, 39].

## 3. Oxidative Damage in Systemic Inflammatory Diseases and AD

### 3.1. Oxidative Damage in the Chronic Inflammatory Disorders

ROS contribute to the progression of chronic inflammatory bone disorders, including RA and periodontitis. The inflammatory cell-mediated overproduction of TNF-*α* is thought to be the main contributor to the increased release of ROS in RA patients [40], because TNF-*α* not only causes cell damage but also inhibits antioxidants, such as superoxide dismutase 1 (SOD1) and SOD3 [41, 42]. Numerous studies have indicated excess ROS levels and the depletion of antioxidant levels in the gingival crevicular fluid [43, 44]. There is further evidence of higher levels of lipid peroxidation, hydrogen peroxides, and oxidative DNA damage in animal models of periodontitis [45]. Indeed, periodontitis is associated with systemic oxidative stress and a reduced global antioxidant capacity, which suggests that oxidative stress in patients with periodontitis could be closely linked to the biomarker of inflammation, including C-reactive protein [46]. It is considered that ROS are involved in the chronic inflammatory bone disorders by regulating osteoblasts and osteoclasts [47], because the increased mitochondria-derived ROS, especially H_2_O_2_, reduces the differentiation and maturation of osteoblasts by inhibiting type 1 collagen and alkaline phosphatase, colony-forming unit-osteoprogenitor formation, and Runt-related transcription factor 2 activation [48, 49]. On the other hand, the increased ROS enhance the osteoclast numbers and resorption by stimulating receptor activator of NF-*κ*B ligand and TNF-*α* expression through extracellular-signal-regulated kinase and NF-*κ*B activation [50].

ROS are increased in the colonic mucosa of patients with the alterations in the mucosal antioxidant defenses in IBD patients [51, 52], because the body's major antioxidant, glutathione, is depleted but its oxidized form, glutathione disulfide, is increased in individuals with active IBD [53, 54]. The imbalance caused by the increase of ROS production and the decrease of antioxidant capacity-induced oxidative stress is considered to be the major pathogenic mechanism of IBD [55, 56]. Excessive levels of ROS result in damage to the cytoskeleton protein, including the temporal disruption of the barrier integrity and increasing gut permeability [57, 58]. Therefore, ROS promote oxidative damage, modulate the intra- and extracellular redox status, and interfere with the activation of proteolytic enzymes in the systemic inflammatory environment.

### 3.2. Oxidative Damage in AD

Oxidative stress is considered to be the main cause of AD. In microglia, mitochondrial dysfunction leads to the excess production of ROS, which promotes the redox imbalance and stimulates proinflammatory gene transcription and the release of cytokines, such as IL-1, IL-6, and TNF-*α*, thereby inducing neuroinflammation. The neuroinflammation-prolonged oxidative stress leads to the accumulation of A*β* and tau phosphorylation and then induces neurotoxicity in AD patients [59, 60]. Thus, microglia-mediated neuroinflammation is perceived as a cause and a consequence of chronic oxidative stress.

Extensive oxidative stress is observed in all of the cellular macromolecules of AD patients. First, lipid peroxidation is greatly enhanced in AD. The 4-hydroxynonal levels are significantly elevated in the hippocampus, entorhinal cortex, temporal cortex, amygdala, parahippocampal gyrus and ventricular fluid [61–64], and plasma [65] of AD patients. Second, the oxidative modification of proteins, which results from either a direct ROS attack or from the reactions that occur through the binding of glycation, glycoxidation, and lipid peroxidation products, has been extensively shown in AD. The most widely studied markers of protein oxidation are protein carbonyls and 3-nitrotyrosine. Significant increases of protein carbonyl are observed in the hippocampus, parietal lobe, and superior middle temporal gyrus of AD patients [66, 67]. Third, oxidative damage occurs in the DNA/RNA of AD patients. High levels of DNA breaks are found in the hippocampus and cerebral cortex of AD patients [68]. 8-Hydroxydeoxyguanosine (8-OHdG) is the most widely used DNA oxidative marker, which is increased in ventricular cerebrospinal fluid [69] and the peripheral tissues, such as sporadic fibroblasts [70], and in the lymphocytes of AD patients [71].

It has been demonstrated that the onset of AD is commonly preceded by an interim phase known as mild cognitive impairment (MCI), when there is no significant increase in senile plaques [72–74]. MCI patients exhibit significant oxidative imbalance in comparison to age-matched controls, since the elevation of overall protein peroxidation and the oxidative modification of specific proteins are detected in the brain, including hippocampus [75, 76], and reduction of the activity of antioxidant enzymes such as superoxide dismutase, glutathione peroxidase, and glutathione is observed in MCI patients [77, 78]. These facts strongly suggest that the oxidative imbalance appears at the very early stage of AD.

Chronic systemic inflammation links to neuroinflammation by the releasing of proinflammatory mediators including IL-1*β* to activate microglia [17, 18]. Repeated LPS-induced chronic systemic inflammation in mice induces microglial activation and prolonged IL-1*β* production by activated microglia [79]. Furthermore, systemic inflammatory challenge in the late gestation of mice increases the deposition of A*β* and tau phosphorylation, which resulted in the impairment of working memory during adult [36].

The four routes by which systemic immune signals can be transmitted to the brain have been intensively studied [17, 18]. In addition to these four classical routes, we have recently found that the leptomeningeal cells, which cover the surface of the brain parenchyma, release the proinflammatory cytokines to activate microglia during systemic inflammatory challenge [80, 81]. Therefore, leptomeningeal cells can transmit signals from systemic immune cells to microglia.

## 4. Microglia Aging Concept: Microglia and Brain Aging

As the phagocytic cells in the brain, microglia are primed during aging, even in middle age. The primed microglia can produce an exaggerated inflammatory response in the brain, because age-dependent dysfunctions of lysosomal/mitochondria system allow for the hypergeneration of ROS. The increased intracellular ROS then activates the redox-sensitive transcription factors, including NF-*κ*B, to provoke exaggerated inflammatory responses [1]. The sensitivity to oxidative stress and activation of redox-sensitive transcription factors during aging may drive the emergence of senescent-type microglia (microglia aging). This may explain why A*β*, which cannot sufficiently activate NF-*κ*B, is able to induce IL-1*β* secretion by activated microglia isolated from the aged mouse brain but not from the young adult mouse brain [82].

It is noted that chronic systemic inflammation induces age-dependent differential responses in microglia. The activated microglia produce anti-inflammatory mediators in young adult adjuvant arthritis (AA) rats, an animal model of RA [18, 80, 81]. However, the activated microglia produced proinflammatory mediators in the middle-aged AA rats [83]. Therefore, chronic systemic inflammation induces microglia aging from middle age. Furthermore, the microglia aging induces the functional outcomes during systemic inflammation. The long-term potentiation (LTP), a cellular substrate involved in learning and memory, in the hippocampus is significantly decreased in middle-aged AA rats but not in young adult rats. The systemic administration of minocycline, a known inhibitor of microglial activation, significantly restores the formation of LTP in middle-aged AA rats. These observations suggest that chronic systemic inflammation induces deficits of learning and memory through microglia aging [84].

Microglia is highly sensitive to excessive ROS activated NF-*κ*B due to the increased oxidative mitochondrial DNA (mtDNA) damage [1]. The hypoxia activates the NF-*κ*B signaling pathway to induce microglia aging [6–8, 11]. Furthermore, microglia are recognized as the major cells for NF-*κ*B-dependent proinflammatory mediators production during stroke, the most common form of hypoxia-ischemic brain injury [85, 86]. The microglia aging mediated neuroinflammatory responses are closely associated with the pathogenesis of AD [12], because the proinflammatory mediators promote neuronal cell damage and excessive A*β* deposition [12, 87]. These observations suggest that the microglia aging is an important causative factor for AD.

## 5. Nutrients in Microglia Aging and Cognitive Function 

### 5.1. Propolis

**Figure 1:**
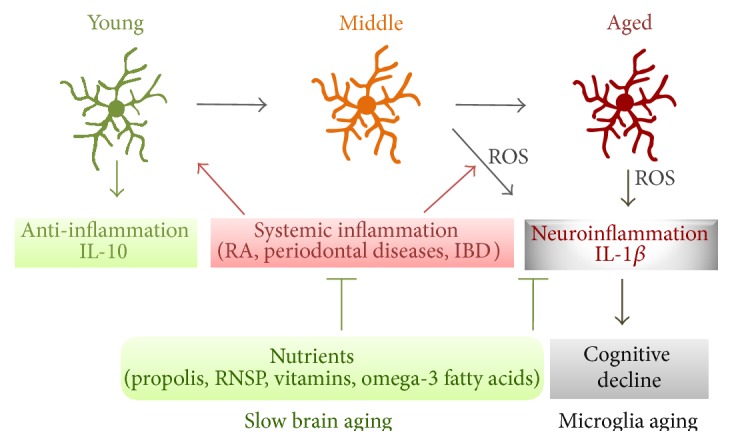
A schematic representation of the preventing and reversal of microglia aging by the antioxidant nutrients. Increased microglial mitochondria-derived ROS induce neuroinflammation which initiates cognitive decline during aging. Chronic systemic inflammation promotes microglia aging even in middle age through excessive neuroinflammation. The oral intake of antioxidant nutrients, including propolis, RNSP, vitamins, and omega-3 fatty acids, will prevent and reverse microglia aging, thereby improving cognitive function and slowing brain aging.

There is increasing evidence that natural nutrients can provide significant benefits in dementia patients [88]. Propolis is a resinous substance which is produced by honey as defense against intruders. It has been used therapeutically since ancient times. The chemical composition of propolis depends on the local floral at the site of collection [89–91]. In addition to the fact that propolis has antioxidative and anti-inflammatory effects [92–94], we recently found that propolis significantly inhibits the secretion of IL-1*β*, TNF-*α*, and IL-6 by microglia by inhibition of the activation of NF-*κ*B signaling pathway [11]. Moreover, propolis was observed to significantly inhibit the increased generation of mitochondria-derived ROS, which is responsible for the activation of NF-*κ*B signaling pathway. Moreover, propolis significantly inhibits the increased expression of 8-OHdG, a biomarker for oxidative DNA damage [95], mainly in the mitochondria of microglia after hypoxia. Since oxidative mtDNA damage impairs the respiratory chain to form a vicious cycle which promotes ROS generation [1], propolis may prevent and reverse microglia aging through its antioxidant property, both systemically and in the brain [92–96] (Figure 1).

### 5.2. RNSP

RNSP is one of the Tibetan medicines composed of 70 natural components. It is used clinically for treating cerebrovascular diseases, cerebral infarction and epilepsy, and brain concussion. Our previous studies showed that RNSP improves learning and memory in a mouse model of AD (Tg2576) [21, 97] and improves the cognitive functions in mild-to-moderate AD patients living at high altitude [98]. Furthermore, RNSP reduces proinflammatory mediators, including IL-1*β*, TNF-*α*, and IL-6, in the activated macrophages and serum in humans, indicating that it also ameliorates the systemic inflammation [98, 99].

### 5.3. Other Nutrients

As a concept which was first introduced in 1985, oxidative stress is used to describe a condition of imbalance between oxidants and antioxidants in favor of oxidants, which potentially leads to cellular damage [100, 101]. Oxidative stress induces the oxidation of DNA, proteins, and lipids. Through detection of 8-OHdG, a marker of oxidative damage to DNA, increased mtDNA damage in the parietal cortex of AD patients was shown, indicating that mtDNA is particularly sensitive to oxidative damage [102]. Furthermore, the intake of antioxidants in patients with MCI was considered to be helpful in lowering the risk of conversion to cognitive impairment because MCI represents a prodromal stage of AD, and oxidative damage appears to occur as one of the earliest pathophysiological events in AD [103, 104].

Studies have shown the roles of the antioxidant nutrients in microglial activation. It was reported that 1,25(OH)_2_D_3_ inhibited the production of TNF-*α*, IL-6, and NO by the stimulated microglia in a concentration-dependent manner, because vitamin D3 receptors are expressed in microglia [105]. Another report showed that vitamin E might provide neuroprotection* in vivo* by attenuating microglial TNF-*α* and NO production by suppressing the microglial activation of p38 mitogen-activated protein kinase and NF-*κ*B [106]. Furthermore, vitamin E reduces the LPS-induced increase in ROS and IL-6 in the primary microglia and the intraperitoneal injection of LPS has been shown to induce lipid peroxidation and IL-6 in the brain [107]. On the other hand, another study showed dramatic microglial activation, particularly in the CA1 region of the hippocampus [108]. More recent research showed that vitamin D deficiency decreases the release of TNF-*α* and IL-6 in cultured microglia upon stimulation with Toll-like receptor agonists [109]. The roles of n-3 fatty acids such as docosahexaenoic acid (DHA) and eicosapentaenoic acid (EPA) in microglial activation have also been reported. It was noted that DHA and EPA decreased the inflammatory responses and increased the anti-inflammatory responses of microglia after the phagocytosis of A*β*42 and that DHA decreased TNF-*α* production, while EPA increased production of brain derived neurotrophic factor in cultured human CHME3 microglial cells [110]. A more recent study showed that DHA and EPA inhibited the release of TNF-*α* and NO from primary microglia which occurs in response to interferon-*γ* and myelin stimulation [111].

A great deal of evidence exists to support the roles of antioxidant nutrients in cognitive function. Vitamin E has been reported to improve cognitive function in elderly individuals [112]. It is known that the soluble A*β* oligomers cause cognitive loss and synaptic dysfunction in AD patients. The treatment with vitamin C for 6 months attenuated A*β* oligomer formation, restored the reduced synaptophysin level, and mitigated the memory behavioral decline in an AD mouse model [113]. More recent research showed that vitamins C and E supplementation mitigated the melamine-induced impairment of hippocampal synaptic plasticity [114]. However, other studies did not find evidence to support the efficacy of vitamin E, B-6, or B-12 as a preventive therapy or treatment in individuals with AD or MCI [115, 116], and 12 months of vitamins E and C supplementation did not improve the mini-mental state examination score of elderly individuals in Iran [117]. The potential role of DHA and EPA in the prevention of cognitive decline, including the decline associated with AD, has attracted major interest over the past 20 years. Recent research showed that n-3 fatty acids supplementation ameliorated memory deficits, which increased the serum total antioxidant capacity [118]. On the other hand, EPA and DHA supplementation for 2 years was not found to affect the cognitive decline in healthy elderly individuals [119]. Further intervention studies with larger study populations should be undertaken to identify the role of antioxidants in the management of cognitive function.

Approaches with multiple antioxidant nutrients to block the oxidative stress related to the systemic and brain inflammation pathways may therefore prevent or delay the cognitive impairment associated with AD by preventing microglia aging.

## 6. Conclusion

We herein provided the concept of microglia aging as a brain aging accelerator, which is associated with cognitive decline during aging and in AD. Chronic systemic inflammation promotes microglia aging even at middle age. Certain nutrients may therefore be beneficial for delaying brain aging by preventing or reversing microglia aging (Figure 1).
